# Chelating Agents Usage in Optimization of Fracturing Fluid Rheology Prepared from Seawater

**DOI:** 10.3390/polym13132111

**Published:** 2021-06-27

**Authors:** Amro Othman, Murtada Saleh Aljawad, Mohamed Mahmoud, Muhammad Shahzad Kamal, Shirish Patil, Mohammed Bataweel

**Affiliations:** 1 Department of Petroleum Engineering, College of Petroleum Engineering & Geosciences, King Fahd University of Petroleum & Minerals, Al Dharan 31261, Saudi Arabia; g201904750@kfupm.edu.sa (A.O.); mmahmoud@kfupm.edu.sa (M.M.); shahzadmalik@kfupm.edu.sa (M.S.K.); patil@kfupm.edu.sa (S.P.); 2 EXPEC ARC, Saudi ARAMCO, Dhahran 31311, Saudi Arabia; mohammed.bataweel@aramco.com

**Keywords:** chelating agents, fracturing fluid rheology, seawater, pH, high temperature and pressure, polymer

## Abstract

Hydraulic fracturing consumes massive volumes of freshwater that is usually scarce and costly. Such operation is not sustainable, and hence seawater could be used as an alternative. Nevertheless, seawater has high total dissolved solids (TDS), affecting the fracturing fluid rheology and providing a damage potential to the subterranean hydrocarbon reservoirs. Resolving these issues requires fracturing fluid systems with many additives, which results in an expensive and non-eco-friendly system. This study proposes eco-friendly and biodegradable chelating agents that could replace many additives such as scale inhibitors and crosslinkers. The study aims to optimize the rheology of seawater fracturing fluids using a chelating agent and polymer. By optimizing *N*,*N*-Dicarboxymethyl glutamic acid (GLDA) conditions, high viscosity was achieved using a standard industry rheometer. The GLDA was mixed with carboxymethyl hydroxypropyl guar (CMHPG) polymer and tested in both deionized water (DW) and seawater (SW). The polymer was examined first, where the rheology did not show a time-dependent behavior. The polymer in SW showed a slightly higher viscosity than in DW. The GLDA and CMHPG were tested at different temperatures, pH, and concentrations. These sets showed a time-dependent viscosity behavior, which can be utilized in various fracturing steps. Results showed that the solution pH and GLDA concentration significantly impacted the fluid viscosity magnitude and behavior. The developed formulation is shear thinning, where the viscosity declines as the shear rate increases. The temperature negatively impacted the viscosity and caused the formulation to break. The study provided an understanding of how to optimize the rheology of SW fracturing fluid based on GLDA chelating and CMHPG polymer.

## 1. Introduction

Hydraulic fracturing processes involve pumping slurry to create fractures in the formation and keep them open under closure stresses. Hydraulic fracturing is implemented to increase well productivity and control sand production. This could be achieved through bypassing the near wellbore damage, extending the fracture to a significant depth within the formation, and alter the flow pattern [1]. The stimulation attempts started in the 1860s with explosive charges [2]. These attempts continued until 1947, where the first successful hydraulic fracturing job was performed in Kansas, USA. Only 2.84 m^3^ (750 gallons) of crude oil or kerosene, 18.14 kg (40 lbs) of sand, and 7.46–11.2 kW (10–15 hydraulic horsepower—HHP) were used in the fracturing operation. After that, water-based fracturing fluids are used instead of oil-based. Surfactants and stabilizers were used for the first time. Crosslinkers were developed and used broadly with a target to improve the fluid viscosity. In the 1980s the horizontal drilling revolutionized the fracturing industry and led to massive fracturing operations with more than 1119 kW (1500 HHP) and 950 m^3^/h (100 bbls/min) pumping rates. In 2012, more than 2.5 million fracturing jobs were performed worldwide; more than 40% of those jobs were in the US [3,4].

In hydraulic fracturing design, the fluid viscosity needs to be optimized. The fracturing fluids should be able to carry the proppant with low settlement velocity and create long fractures. It should have the minimum pressure losses when transported in the tubing and fractures, low fluid loss, and ready to flow back after proppant placement (ready to break). It should also be compatible with formation fluids [5,6]. Based on the formation type, government and environment regulations, budget, and water availability, different types of hydraulic fracturing fluid can be used. These fracturing fluids are based on water, oil, alcohol, and foams. The water-based fracturing fluid includes crosslinked fluid, slickwater fluid, and viscoelastic surfactant, while the oil-based can be crosslinked and emulsified [7]. The crosslinked gel has high proppant transport capacity and distribution efficiency. The disadvantages of the gel are the need for high pumping pressures and its damaging impact. Slick water needs lower power to pump the fluid, is cheaper than the crosslinked fluid, and it provides lower damaging impact. However, it has lower transport capacity and poor distribution of the proppant. The foam fluids can carry the proppant better than slickwater; it consumes less water and reduces the formation damage. On the other hand, it has poor suspension and distribution for the proppant when high reservoir temperatures are experienced [8].

The gelling agents are hydrated—mixed with water—to prepare the linear gel fluid, which is used as a pre-pad (lead fluid) to condition the formation and initiate the fractures. The guar derivative powders are prepared by exposing the guar to a high pH water for a certain time. Different polymers provide different leak-off properties to the formation. Guar is a long-chain polymer with a high molecular weight [9]. A crosslinker is added to the linear gel to help increase the viscosity and propagate the fracture. This crosslinked fluid is also used to transport the proppant into the fracture [10]. Adding crosslinkers to the gelled fluid increases the viscosity and reduces the needed concentration of polymer, which raises the fracturing fluids carrying capacity. Several types of crosslinkers are used based on the needs. After proppant placement, the flow back is necessary; hence, breakers must also break these crosslinked fluids [9]. One of the robust methods to investigate the properties of the required fluids is through numerical simulations [11,12]. In this approach, the needed fracturing fluids can be screened based on the reservoir conditions and required properties.

Optimizing hydraulic fracturing operations to use less freshwater and toxic additives became a necessity. Using water-based fracturing fluid has some limitations such as high cost, huge water consumption, formation damage, and costly disposal [13,14,15]. Freshwater is mostly used in fracturing operations; nevertheless, it is an expensive and unsustainable option. Seawater is another alternative that is cheap and sustainable. The high salinity of seawater could result in formation damage and viscosity degradation. Several additives could be used to reduce the impact of different ions exist in seawater. In freshwater or seawater, fracturing fluids might contain gelling agents, crosslinkers, breakers, surfactants, scale inhibitors, corrosion inhibitors, clay stabilizers, biocide/bactericide, fluid loss inhibitors, chelating agents, pH modifiers, and acids [16].

**Figure 1:**
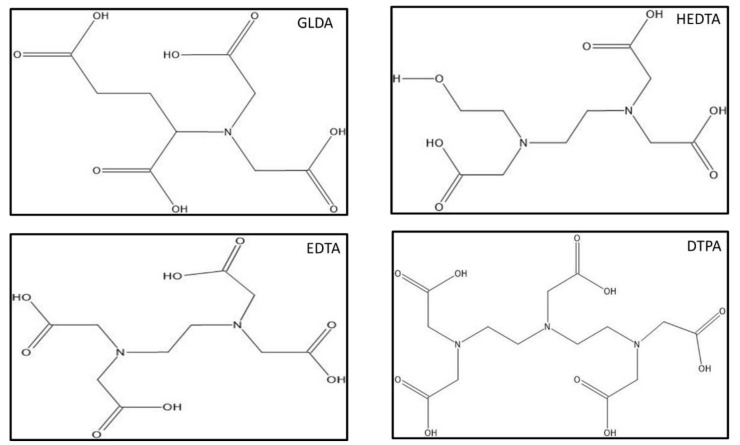
Chemical structure of different chelating agents.

Several types of chelating agents are used in the industry, such as Ethylenediaminetetraacetic acid (EDTA), Diethylenetriamine pentaacetate acid (DTPA), Ethanolic phosphotungstic acid (EPTA), and l-glutamic acid-*N*,*N*-diacetic acid (GLDA), Hydroxyethylethylenediaminetriacetic acid (HEDTA). Figure 1 [17] shows the chemical structure of different chelating agents used in the industry.

Chelating agents are used to replace many additives such as crosslinkers, breakers, and biocides. Different chelating agents have been tested with several polymers (i.e., HPG, Xanthan, Thermoviscofying polymer) to inspect the rheology and the fluid stability in HPHT conditions. A study showed that the high pH GLDA, DTPA, and EDTA chelating agents act as crosslinker. However, only the GLDA breaks at high temperatures [18].

**Figure 2:**
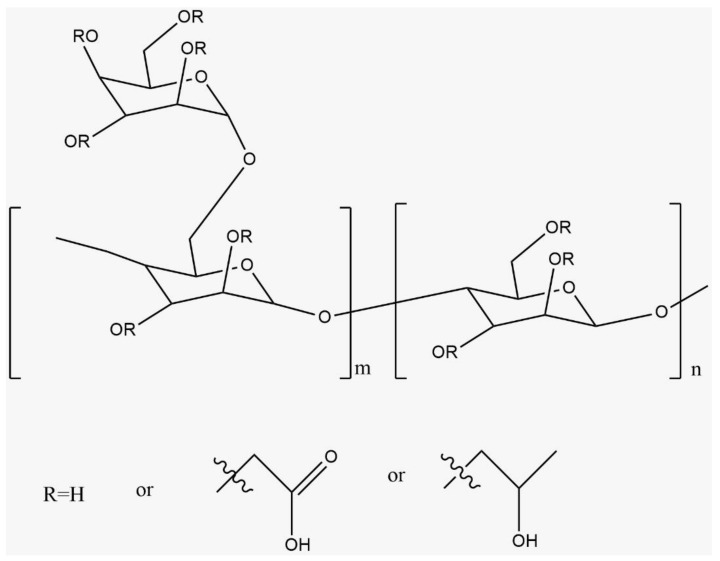
CMHPG generic structure [25].

The two main additives utilized in this study to improve the seawater (SW) rheology are polymer and chelating agents. The gelling agent is the most important additive in fracturing fluids used to increase the water viscosity. High viscosity leads to efficient hydraulic fracturing, high proppant carrying capacity, wider fractures, and reduced fluid loss to the formation. Higher viscosity can be obtained by increasing gel loading, which is the volume of the gel used (in gallons) per thousand gallons of water (gpt). Polymers for fracturing purposes are water-soluble and could come from natural sources such as guar. Chemical modification is applied to natural polymers to achieve the desired physical properties. Carboxymethyl hydroxypropyl guar (CMHPG) or carboxymethyl hydroxyethyl cellulose guars (HEC) are examples of modified guar derivatives. They are desired and widely used because of their low cost and ease of hydration. The low gel load of these polymers could achieve high viscosity and proppant transport [7,9,16,19]. Natural guar polymers have high shear stability and better clean-up compared to other systems [20]. Recent advances in the polymerization process have been reviewed in terms of polymer interactions, polymerization mechanisms, and techniques used in the industry [21]. CMHPG is created by dilution of native guar in a specific solution to get a more rigid chain. CMHPG guar derivative is preferred in the industry for its better hydration, slower degradation, and tolerance to a low pH environments. Figure 2 shows the generic structure of CMHPG ([22,23,24,25]).

Different guar networks can be generated in CMHPG either using hydroxyl group with borate or carboxyl group with metal, i.e., zirconium. CMHPG is reversibly crosslinked by borate, which allows the proppant to be suspended for longer times at high shear rates. While zirconium irreversibly crosslinked with CMHPG where the minimal proppant settling is noticed at low shear rates [26].

There are two methods to crosslink guar derivative, reversible and irreversible crosslinking, depending on the expected shear rate of the operation. Under specific catalysts, several polymer types can be produced with the desired properties; one of these catalysts is introduced by Hanifpour et al. [27]. The constitutional structure of the reversible polymers can be changed using these catalysts; once it is removed, the stability as irreversible polymers can be retained. The irreversible polymer’s stability comes from the stronger bond energy than the reversible polymer [28,29].

The second additive is the GLDA chelating agents (l-glutamic acid-*N*,*N*-diacetic acid). Chelating agents were initially used to reduce formation damage caused by filter cakes and scales around the perforation. Recently, chelating agents were used as a replacement for breakers, iron controls, and viscosifiers such as surfactants, polymers, and cross-linkers [30]. Chelating agents are used with seawater-based fracturing fluids to remove the impact of seawater by removing its hardness [31]. The water hardness impact polymers’ performance and damages the formation. Chelating agents can also be used in improving the thickening effect besides polymers and crosslinkers. The excess concentration of chelating agents can impact the breaker and crosslinker; it is also required to use the minimum possible to remove seawater hardness.

This study focused on GLDA application to SW. GLDA as a chelating agent proved to work as a replacement of crosslinkers, breakers, biocide, clay stabilizers, and HCl. It reduces the interfacial tension of fracturing fluid, compatible with SW, and shows stability at high-temperature environments up to 150 °C [1,32,33]. It has different reaction mechanisms with minerals at different pH levels [30]. It is also biodegradable, which makes it environmentally superior to the other chelating agents.

**Figure 3:**
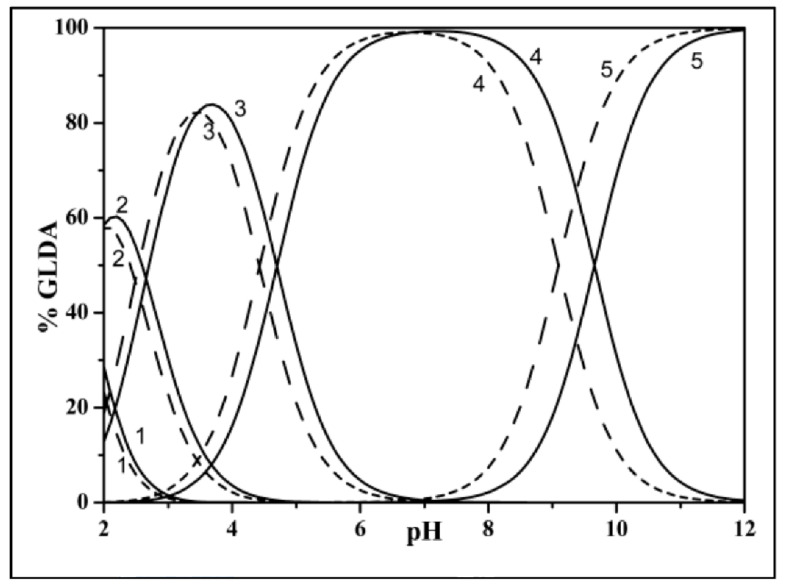
Speciation diagram for GLDA (L) in NaCl(aq) at I = 0.1 mol·kg^−1^ (solid line) and 0.5 mol·kg^−1^ (segment line); cL = 0.01 mol·dm^−3^ 1 = H_4_L; 2 = H_3_L; 3 = H_2_L; 4 = HL; 5 = Lfree Reperinted with permission from (Bretti et al., 2016) copyright (2021) American Chemical Society [34].

To prepare GLDA chelating agent, the speciation chart is used. Speciation determines the equilibrium distribution of chemical species in aqueous solutions. Chemical equilibrium methods are used to determine this distribution (i.e., finding the degree of dissociation constant). In the speciation curves, the percentage of the ions within the compounds are plotted against the pH level. Each curve has a specific number of hydrogen ions. At a specific pH, the GLDA will be formed of several species with different hydrogen atoms. Figure 3 shows the speciation curves for GLDA in NaCl (aqueous solution) for several ionic strengths.

Many challenges face freshwater usage in hydraulic fracturing due to the high cost, scarcity of freshwater, and transportation difficulties to offshore platforms. Hence, the oil and gas industry showed a great interest in utilizing seawater (SW) instead of freshwater in hydraulic fracturing operations. Two types of ions are abundant in SW: divalent (Ca, Mg) and monovalent (Na, K) ions; these ions affect the fracturing fluid rheology and could damage the formation. The monovalent ions only affect the rheology when divalent ions are absent [35]. At a pH greater than 10, we can expect the precipitation of Ca and Mg ions, forming different scales. Therefore, scale inhibitors should be used, and pH should be controlled to avoid scale precipitation. SW thermal stability should also be monitored carefully [36].

In this research, gelling chelating, polymer, and pH adjusting agents were tested with SW. The solution rheology was tested at different conditions (i.e., concentrations, pH, temperature, pressure, shear rate), aiming to identify the optimum. This study shows the impact of GLDA chelating on the rheology of seawater. Noting that the standard GLDA used in this study (high and low pH) is 40% active concentration of the used GLDA solution. It also illustrates how the pH level could be adjusted to obtain the desired viscosity behavior.

## 2. Materials and Methods

The focus of the study is to optimize chelating agents’ concentration and pH to accomplish high viscosity utilizing the competent industry rheometer (Anton Paar-MCR 302), provided by Anton Paar company, Graz, Australia. SW-based fracturing fluid was formulated by adding CMHPG polymer and GLDA chelating agents to SW. Different pH levels (adjusted with sodium hydroxide (NaOH)), polymer concentrations, and chelating agent concentrations were tested at standard conditions and at high pressure and high temperature (HTHP) (i.e., 75–100 °C and 3.45 MPa). All experiments were performed at a constant shear rate of 511 1/s to assess the viscosity behavior versus time. At 10 wt% GLDA and 8.85 pH, different shear rates (100 1/s, 171 1/s, 511 1/s, and 1022 1/s) were tested to evaluate the shear impact on the viscosity magnitude. Anton Paar rheometer was used for most of the tests using a concentric cylinder for experiments at ambient conditions, and the pressure cell was used for higher temperatures. The concentric cylinder volume was 20 mL, while the pressure cell volume was 14 mL. First, to provide a benchmark for the experiments, the CMHPG polymer was tested as a standalone additive with 0.5 wt% and 1 wt% concentration in seawater (SW) and deionized water (DW). This test was performed at ambient conditions and then at HPHT. The composition of SW used in this research is shown in the following Table 1.

Following the baseline tests, the GLDA was tested at both ambient and high temperatures. Two types of GLDA provided by the Nouryon company, Dammam Saudi Arabia (both were 40% active of the provided liquid solution) were used:

1. A low pH GLDA (pH = 4) where NaOH was added to raise the pH gradually from 4 to 10.
2. A high pH GLDA as received from the manufacturer, the pH measured was 13.7, without NaOH addition.

The pH was recorded using two methods, OAKLON pH2700 pH meter, and litmus papers. Next, employing only the high pH GLDA, different concentrations were used to prepare the samples: 1 vol%, 3 vol%, 5 vol%, and 10 vol% and test its rheology at standard conditions. The base GLDA pH was 13.7, but the prepared solution with SW and CMHPG polymer with different GLDA concentrations altered the solution pH level. 1 vol% concentration of GLDA resulted in 10.2 pH, 3 vol% resulted in 10.7 pH, 5 vol% GLDA concentration resulted in pH = 11, and 10 vol% resulted in 11.8 pH. The experiments in this research can be divided as follows:

### 2.1. Concentric Cylinder Experiments (Standard Conditions)

A baseline testing of the polymer potential without GLDA, 0.5 wt%, and 1 wt% CMHPG polymer was conducted with DW and SW at standard conditions for 8 h. The following experiments were performed for 48 h with SW to test the hydration for 0.5 wt% of CMHPG and 10 vol% of GLDA at 5 different pH levels between (4–10). First, the specific pH GLDA was added to SW, where CMHPG polymer was added gradually and mixed for 10 min. The mixture was left for another 10 min inside the concentric cylinder as a procedure to equilibrate the temperature. The viscosity of all five samples was measured for 48 h at a 511 1/s shear rate. Similar series of experiments were performed with high pH GLDA (10–13) where NaOH was added. Polymer and GLDA concentration of 0.5 vol% and 10 vol% were used, respectively. Similar preparation procedure, waiting time, and shear rate were applied. To reach equilibrium in viscosity values, the experiments were performed for 72 h. This set of experiments showed the impact of polymer concentration, chelating pH, and water type on the formulation rheology. The shear rate impact on rheology was also tested.

### 2.2. Pressure Cell Experiments (HPHT)

The second set of experiments was performed at HPHT with 0.5 wt% CMHPG polymer and high pH GLDA, where no NaOH was added. 1 vol%, 3 vol%, 5 vol%, and 10 vol% of high pH GLDA (13.7) were used to prepare the SW-based solution, then 0.5 wt% CMHPG was added at 100 °C and 3.45 MPa. At different temperature (25 °C, 75 °C, and 100 °C), a 3 vol% GLDA was added to 0.5 wt% CMHPG. The same standard preparation method, equilibrium time, and shear rate were used in all the experiments. This set of experiments showed the impact of temperature and GLAD concentration on the formulation rheology.

More than 40 experiments were performed in this research, excluding the repeated ones. Table 2 shows a summary of performed experiments in both ambient conditions and HPHT. It shows a summary of the comparison between ambient and HPHT conditions.

## 3. Results and Discussions

This section shows the impact of CMHPG polymer with and without GLDA at different conditions on the formulation rheology. The impact of GLDA pH and concentration at different temperatures were examined.

### 3.1. pH Control

**Figure 4:**
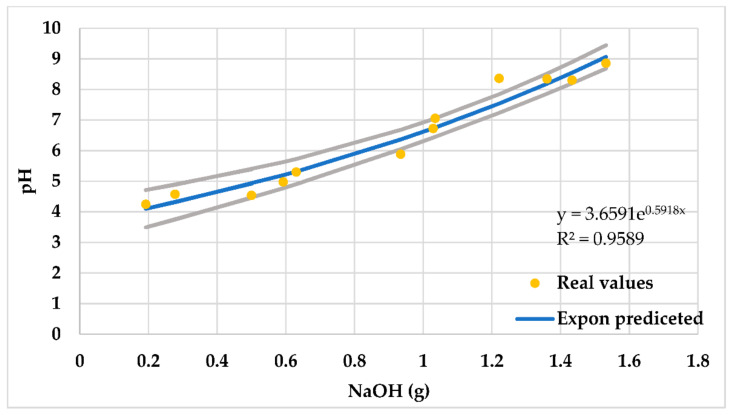
Effect of adding NaOH on chelating agent pH level when 10 vol% added to SW.

**Figure 5:**
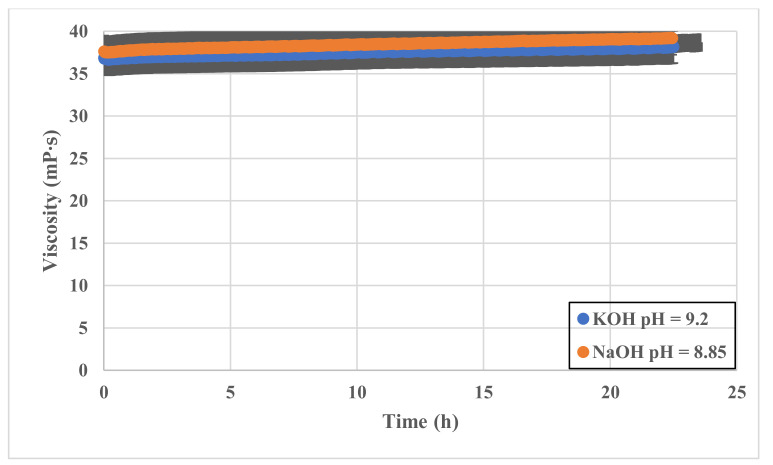
Rheology comparison for different pH controllers added to GLDA.

Two GLDA types were obtained from the manufacturer: one is low pH (4) and the other is high pH (13.7). As mentioned before, the low pH GLDA solution was raised from pH of 4 to higher values using NaOH. Figure 4 shows a relation between the added mass of NaOH and GLDA pH level. The figure shows that as the mass of NaOH increases, the pH level increases. An exponential function was used to predict the pH level from the added mass of NaOH. The figure also shows the bound of the 95% confidence interval. This positive correlation can be employed to find the required NaOH mass to get the desired pH level. NaOH and KOH were used to raise the pH level of GLDA from 4 to 9. This is to investigate if the type of salt used to increases the pH has any impact on the rheology. Both showed a similar effect on the rheology of the SW at similar pH, as the viscosity values and trend were similar (Figure 5). The black backgrounds in Figure 5 and the subsequent ones are the error bars. Since the data points of each curve are more than 1000, the bars form this black background. These bars show the variability of the plotted data; for this reason, we see the black background of curves thicker than other parts in some parts of the curves, similar method was used in [37].

### 3.2. GLDA pH Impact on Viscosity

**Figure 6:**
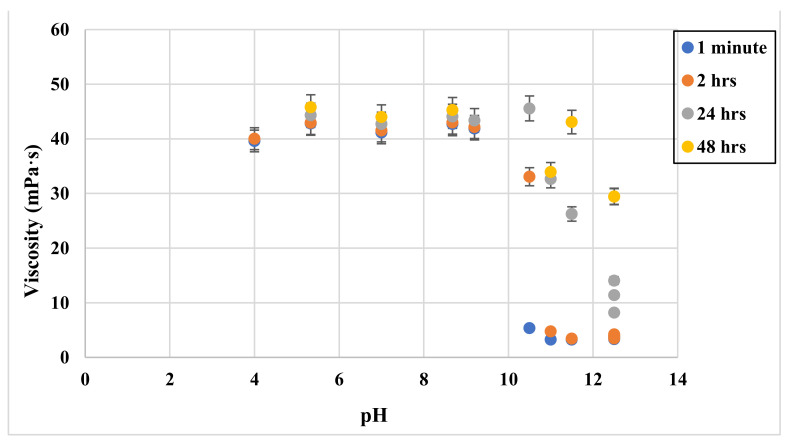
Viscosity versus GLDA pH at 1 min (blue circle), 2 h (red circle), 24 h (black triangle), and 48 h (yellow square).

GLDA solutions at different pH levels were added to SW and mixed with 0.5 wt% CMHPG polymer. The viscosity of the solution was tested at standard conditions. Figure 6 shows how the GLDA pH affects the viscosity of the solution at 1 min, 2 h, 24 h, and 48 h of shearing, respectively. The values of the first 2 h of shearing (blue and red circles) show the big difference in viscosity between the high and low pH samples. We can notice from the figure that as the pH exceeds 10, the viscosity of the solution drops sharply. While after shearing for a long time (i.e., 48 h), the viscosity of the high pH samples increases sharply. On the other hand, the samples’ viscosity when the pH is lower than 10 stayed almost constant even after shearing for 48 h.

### 3.3. Prolong Viscosity Tests

#### 3.3.1. Standard Conditions

At standard conditions, the viscosity along shearing time was acquired from the rheometer, which was performed at a shear rate of 511 1/s. First, DW and SW were tested as a base case with only CMHPG polymer (i.e., in the absence of GLDA). The results indicated that the DW-based fluid has a slightly higher viscosity as compared to SW. In general, the water type did not impact the viscosity magnitude within 8 h of shearing.

**Figure 7:**
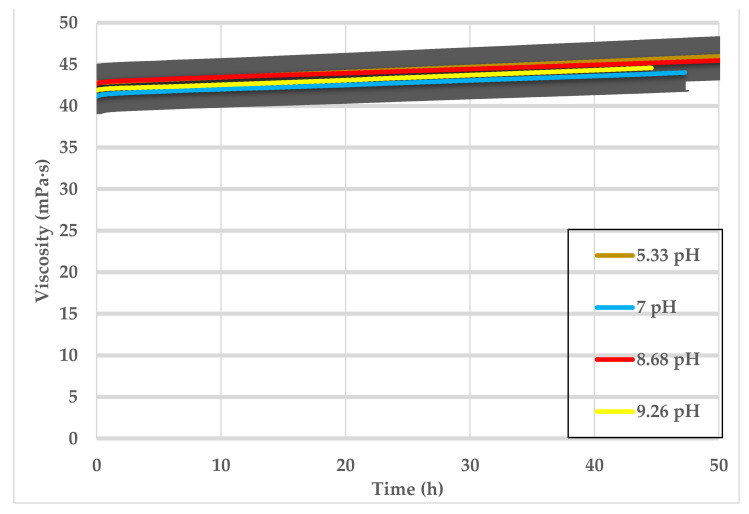
SW based viscosity versus time, 0.5 wt% CMHPG polymer mixed with low pH values of GLDA < 10.

Different pH solutions 4 < pH < 10 were obtained by adding NaOH to low pH GLDA. These solutions were used to prepare different SW-based samples and tested at ambient conditions. The selected concentration of CMHPG polymer and GLDA were 0.5 vol% and 10 vol%, respectively. The tested viscosities for these samples are shown in Figure 7. It clearly shows that increasing the pH from 4 to 10 of the low pH GLDA by adding NaOH did not impact the viscosity magnitude or trend.

**Figure 8:**
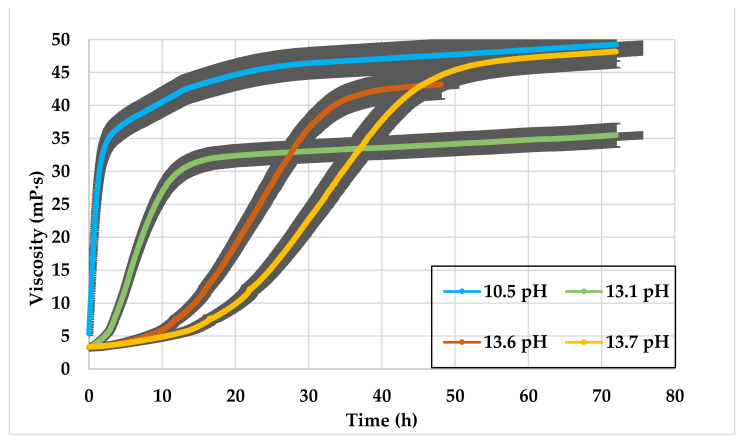
SW based viscosity versus time, 0.5 wt% CMHPG polymer mixed with 10 vol% high pH GLDA > 10.

The GLDA solutions with pH higher than 10 were prepared using NaOH from the low pH GLDA. In all cases, SW is mixed with 10 vol% of GLDA and 0.5 wt% CMHPG polymer at standard conditions. The results showed that the viscosity starts at very low values (3 mPa· s^−5^ mPa·s) and reaches high values after shearing for a couple of hours. At late shearing times, the high pH GLDA viscosity increases gradually, showing similar behavior to those in Figure 7. As the pH of the GLDA increases, the increase in viscosity takes longer durations to appear, as illustrated by the curves turning points shown in Figure 8. The GLDA pH level might impact the speed of polymer hydration, and hence the time-dependent behavior appeared.

**Figure 9:**
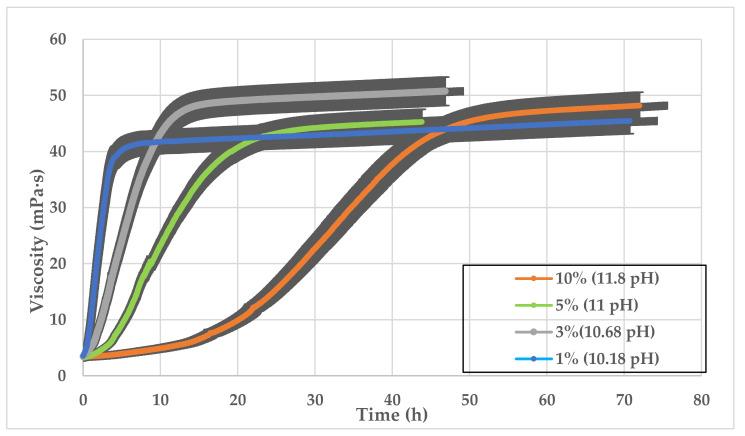
SW based viscosity versus time, 0.5 wt% CMHPG polymer at high pH values of GLDA (13.7) with different GLDA concentrations.

The high pH GLDA provided by the company was tested to find the most practical concentration. It is desirable to use the lowest GLDA concentration that will still reduce SW hardness. GLDA of 1 vol%, 3 vol%, 5 vol%, and 10 vol% concentrations were added to SW and mixed with 0.5 wt% CMHPG polymer. The viscosity versus time of the tested solutions is shown in Figure 9. The figure indicates that the higher the GLDA concentration, the longer time it takes to achieve high viscosity. This is similar to the pH behavior shown in Figure 8, which could be attributed to delayed hydration of polymer. It can also be noticed from this set of experiment, that the 1 vol% concentration of high pH GLDA (13.7) has similar behavior to the lower pH (10.5) 10 vol% GLDA (see Figure 8 and Figure 9). This shows that the higher pH GLDA with lower concentration could give the same viscosity trend and value to the higher concentration lower pH GLDA. High GLDA concentration or pH might be desirable if a delayed viscosity increase is needed.

**Figure 10:**
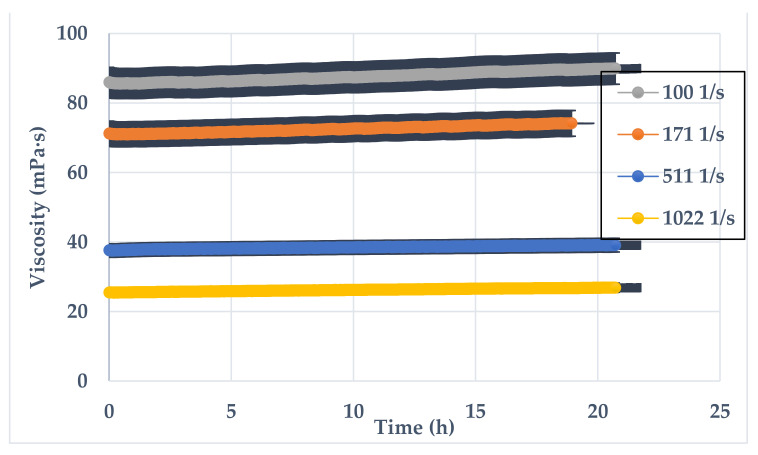
SW-based viscosity versus time, 0.5 wt% CMHPG polymer 10 vol% of GLDA with (pH = 8.85) at standard conditions and different shear rates.

To test the shear rate impact, 10 vol% GLDA with pH = 8.85 was added to SW and 0.5 wt% polymers at standard conditions. The tested shear rates were 100 1/s, 171 1/s, 511 1/s, and 1022 1/s which are industry standard. Figure 10 shows that as the shear rate increases, the viscosity values decrease, showing a shear-thinning behavior.

#### 3.3.2. HPHT Conditions

**Figure 11:**
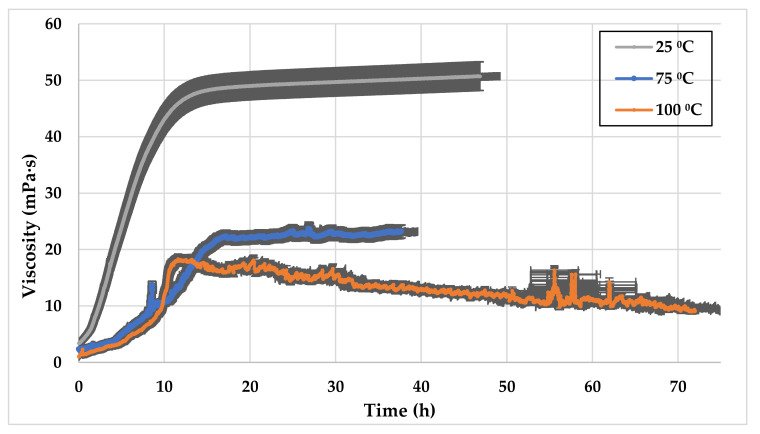
SW based viscosity versus time, 0.5 wt% CMHPG polymer 3 vol% high pH GLDA (13.7) at different temperatures.

In this set of experiments, a 3 vol% of the standard high pH GLDA (13.7) was mixed with SW and 0.5 wt% CMHPG. Three samples were prepared and tested at 25 °C, 75 °C, and 100 °C. All experiments were performed at pressure cells of 3.45 MPa and a shear rate of 511 1/s. The viscosities of samples at the different temperatures are compared in Figure 11. It is noticed that the higher the temperature, the lower the viscosity magnitude. The thickening effect breaks at less than 15 h when the temperature is 100 °C.

**Figure 12:**
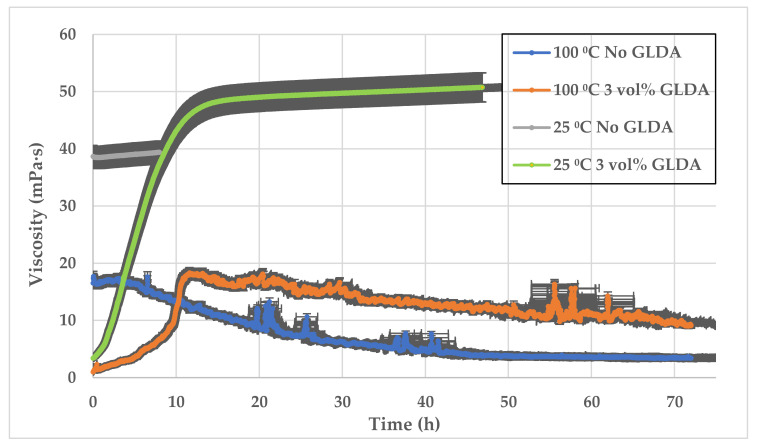
SW-based viscosity versus time, 0.5 wt% CMHPG polymer with and without 3 vol% high pH GLDA (13.7) at two temperatures 25 °C, and 100 °C.

Figure 12 shows the impact of GLDA on formulation rheology at different temperatures. At 25 °C, the 3 vol% GLDA and no GLDA samples (all are SW-based mixed with polymer) were tested and compared. The tests were at 100 °C, as shown in Figure 12 The viscosity starts at higher points when no GLDA is added to the solution in both low and high temperatures. The figure indicates that after shearing, the GLDA samples provided higher viscosity at high and low temperatures. It clearly shows the thickening effect of GLDA when added to CMHPG polymer.

## 4. Conclusions

This paper discusses the rheology of SW-based fracturing fluid when the CMHPG polymer and GLDA chelating agent are the only additives. The water type (SW vs. DW) did not impact the rheology of CMHPG polymer even after 8 h of constant shearing. The formulation suggested in this research can replace freshwater, cut transportation cost in offshore operations, and reduce operating costs. We observed that the formulation viscosity and the hydration time could be designed by either varying the GLDA concentration or pH value. The higher the concentration and pH value, the longer it takes for the polymer to hydrate and the viscosity to increase. This control can be used to reduce the pumping power necessary to transport fracturing fluid into the formation. GLDA chelating agent improved the performance because it captures the SW ions and reduces its hardness, which reduces the formation damage due to SW usage. The combination of CMHPG polymer and GLDA chelating provide a thickening effect which is reduced at 100 °C. The suggested formulation is considered eco-friendly and biodegradable.

## Figures and Tables

**Table 1:** SW formulation used in the experiments.

Compound (Anhydrite)	g/L
NaHCO_3_	0.165
Na_2_SO_4_	6.339
NaCl	41.172
CaCl_2_.2H_2_O	2.387
MgCl_2_.6H_2_O	17.644
Total dissolved solids (TDS)	67.707

**Table 2:** Rheology experiments performed at standard and HPHT conditions.

Ambient Conditions	HPHT	Comparison
DW with 0.5 wt% and 1 wt% CMHPG (Baseline test) SW with 0.5 wt% and 1 wt% CMHPG (Baseline test)	SW with 0.5 wt% and 1 wt% CMHPG (100 °C) (Baseline test)	DW vs. SW Temperature impact on rheology
**pH tests**	**Temperature tests**	
10 vol% GLDA (4–13.7 pH) and 0.5 wt% CMHPG 1 vol%–10 vol% GLDA (13.7 pH) and 0.5 wt% CMHPG	3 vol% GLDA (13.7 pH) and 0.5 wt% polymer (25 °C, 75 °C, 100 °C)	GLDA impact at ambient conditions GLDA impact at different temperatures
